# Supplementary material for: Microfluidic flow-cell with passive flow control for microscopy applications
Source: PLoS One. 2020 Dec 15;15(12):e0244103. doi: 10.1371/journal.pone.0244103 (PMC7737957; doi:10.1371/journal.pone.0244103)
Supplement: Attachment — Submitted filename: Reviewer response 111120.docx [file pone.0244103.s002.docx]

Reviewer #1: In this manuscript the authors reported the design of a lab-fabrication friendly flow chamber. Such flow chamber can be prepared without the requirement of microfabrication facilities, or other workshop instruments. Overall the authors did a great job describing the detailed design concept regarding the chamber, and the following proof-of-concept application study is well planned. However, there are still several issues that authors need to address, and some additional information needed to support all their claims. Based on its current status, I would recommend “revision”.

We thank the reviewer for his/her comments which have helped to strengthen the paper.

1) The author claimed that the chamber can be made in a high-throughput fashion, but didn’t provide much support to demonstrate the chamber to chamber variability, results reproducibility, etc. and how much “faster” can it be when compared to the PDMS lithography based technologies?

We have added a new figure – Figure 5 – where we develop a device with multiple channels and measure the variation from nineteen channels in five devices using a flow rate sensor. This demonstrates high reproducibility with only a 5% variation in flow rate between channels (IQR/median).

Figure 5 – Multiple channel design and reproducibility test. (A-E) Key steps in fabricating the flow chamber. The steps are carried out left to right in two rows. (F) Image of flow chamber filled with four different dye solutions. (G) Flow sensor recordings from four channels on one device. Each channel was filled with deionised water and 30µL deionised water was added at t=10s. (H) Flow rate values measured for 19 channels across five devices (one channel was blocked with PDMS and therefore not measured).

It is not straightforward to answer exactly how much faster this technique is compared to PDMS lithography. It depends to some extent on the skill of the experimenter and what they are seeking to do in the experiment. It is certainly faster to make new tape cut channel designs since this only requires a vector graphics design to be sent to the programmable cutter whereas PDMS microfluidics requires multiple steps including photomask fabrication, spin-coating, UV exposing, developing and plasma bonding. However once a PDMS master has been made then it is fairly simple to make multiple copies of a device quickly. Given these different factors, we prefer to avoid giving a strong statement on this and have amended our sentence in the manuscript:

“The method is high-throughput since it only requires simple benchtop apparatus and does not require glass drilling, complex machining or lithography.”

to

“The method requires only simple benchtop apparatus without the need for glass drilling, complex machining or lithography.”

2) How does the chamber perform when compared side-by-side with a similarly designed lithography designed microfluidic chamber? What’s the novelty of the chamber performance-wise?

There are several aspects of novelty of this method over PDMS lithography. We have added several sentences at the end of the paper to compare and contrast our method with traditional lithographic approaches.

“In contrast to PDMS lithography-based microfluidics, our method enables rigid, ultrathin devices. These flow cells therefore have particular application in microscopy studies which require close access to the sample e.g. with high numerical aperture objectives and condenser optics or for techniques such as magnetic and optical tweezers. Since the contact angle for water on glass is significantly lower than water on PDMS, the use of glass for the top and bottom layers of the device also enables strong capillary stop valves. The (x,y) resolution of the channels formed using adhesive tape is, however, lower than what is possible with lithography-based microfluidics. PDMS lithography standardly enables feature sizes down to the micron level^19^. From our experience, the minimal channel width that can be fabricated, in adhesive tape, with a programmable mechanical cutting blade is ~500µm. A recent demonstration showed that a laser cutter can be used to make channels in adhesive tape with widths down to ~100µm^20^.”

To emphasise the fact that we are able to achieve passive flow and passive stopping of the flow with this technique, which to our knowledge is a novel aspect in flow cell design, we have amended the title of the paper to

“Microfluidic flow-cell with passive flow control for microscopy applications”

3) Would the material difference between the chamber wall material (the tape) and the top/bottom material (glass) create any change in the flow pattern? And how would the different types of the tape material impacts the flow?

The tape comprises a very small amount of the surface area of the channel and therefore should not impact the flow profile which is dominated by the glass surfaces. To emphasise this, we have added a new device cross-section in Figure 1 (Figure 1I) which is to scale.

We also added a photograph of the device in Figure 2 (Figure 2a) to help illustrate the geometry of the experimental setup.
